# The Relationship Between Local Field Potentials and the Blood-Oxygenation-Level Dependent MRI Signal Can Be Non-linear

**DOI:** 10.3389/fnins.2019.01126

**Published:** 2019-10-25

**Authors:** Xiaodi Zhang, Wen-Ju Pan, Shella Keilholz

**Affiliations:** The Wallace H. Coulter Department of Biomedical Engineering, Georgia Institute of Technology, Emory University, Atlanta, GA, United States

**Keywords:** local field potentials, BOLD, electrophysiology, fMRI, non-linearity, neurovascular coupling, correlation

## Abstract

Functional magnetic resonance imaging (fMRI) is currently one of the most important neuroimaging methods in neuroscience. The image contrast in fMRI relies on the blood-oxygenation-level dependent (BOLD) signal, which indirectly reflects neural activity through neurovascular coupling. Because the mechanism that links the BOLD signal to neural activities involves multiple complicated processes, where neural activity, regional metabolism, hemodynamics, and the BOLD signal are all inter-connected, understanding the quantitative relationship between the BOLD signal and the underlying neural activities is crucial for interpreting fMRI data. Simultaneous local field potential (LFP) and fMRI recordings provide a method to study neurovascular coupling. There were a few studies that have shown non-linearities in stimulus related responses, but whether there is any non-linearity in LFP—BOLD relationship at rest has not been specifically quantified. In this study, we analyzed the simultaneous LFP and resting state-fMRI data acquired from rodents, and found that the relationship between LFP and BOLD is non-linear under isoflurane (ISO) anesthesia, but linear under dexmedetomidine (DMED) anesthesia. Subsequent analysis suggests that such non-linearity may come from the non-Gaussian distribution of LFP power and switching from LFP power to LFP amplitude can alleviate the problem to a degree. We also confirmed that, despite the non-linearity in the mean LFP—BOLD curve, the Pearson correlation between the two signals is relatively unaffected.

## Introduction

After its inception in the early 1990s (Ogawa et al., 1990, 1992; Belliveau et al., 1991), functional magnetic resonance imaging (fMRI) quickly became the dominant method to study brain activity. Later on, Biswal et al. (1995) found that the fMRI acquired without a task reveals synchronous fluctuations in different brain regions, which reflects the functional connectivity. Whether the fMRI is performed with task-rest block design, or is performed at rest without any explicit task, ultimately all fMRI studies rely on a contrast mechanism called blood oxygenation level-dependent (BOLD), in order to non-invasively detect the relative neural activity. The image contrast in BOLD fMRI comes from the fact that deoxyhemoglobin is strongly paramagnetic, whereas oxyhemoglobin is diamagnetic. The presence of paramagnetic deoxyhemoglobin distorts the local magnetic fields, and such distortion results in intravoxel dephasing of MRI signal (T2* weighting), which reduces the signal intensity in that region (Thulborn et al., 1982; Silvennoinen et al., 2003). Since the brain does not store oxygen, an increase of neural activity will demand more oxygen, and through a process called neurovascular coupling, the regional cerebral blood flow (CBF) will increase to fulfill the demand (Fox and Raichle, 1986). This brings more oxygenated blood to this region and lowers the relative concentration of deoxygenated hemoglobin, increasing the BOLD signal. It can be seen that the coupling between neural activity and BOLD signal changes involves multiple processes, and that neural activity, regional metabolism, hemodynamics, and the BOLD signal are all inter-connected via signaling pathways that are not completely understood. The fact that the BOLD signal is only an indirect measurement of neural activity makes the interpretation of fMRI studies difficult (Bandettini and Ungerleider, 2001; Arthurs and Boniface, 2002; Heeger and Ress, 2002; Logothetis, 2008).

To better understand the relationship between BOLD and neural activity, it is necessary to utilize modalities that can directly measure neural activity simultaneously with fMRI. Logothetis et al. (2001) pioneered the development of simultaneous acquisition of local field potentials (LFP) and fMRI data in primates. Their study showed that both LFPs and multi-unit activity (MUA) are correlated with the BOLD response, LFPs showed higher correlation than MUA. This outstanding work not only provided invaluable insights into the relationship between neural activity and the BOLD signal, but also established a feasible method for recording fMRI signal and neural activity simultaneously (Bandettini and Ungerleider, 2001; Arthurs and Boniface, 2002; Heeger and Ress, 2002). As a result, an increasing number of studies have employed simultaneous LFP and fMRI data acquisition (Shmuel et al., 2006; Huttunen et al., 2008; Shmuel and Leopold, 2008; Murayama et al., 2010; Mishra et al., 2011; Pan et al., 2011, 2013; Devonshire et al., 2012; Magri et al., 2012), improving our understanding of the underlying mechanisms that link the BOLD signal to neural activity.

Most studies that analyze the LFP-BOLD relationship, either implicitly (Shmuel et al., 2006; Huttunen et al., 2008; Murayama et al., 2010; Pan et al., 2011, 2013) or explicitly (Logothetis et al., 2001), assume a linear relationship between LFP and BOLD. For example, the Pearson correlation coefficient, the most widely used metric, implicitly assumes a linear dependency. However, the linear model may sometimes be overly simplified, and its applicability remains a topic of debate (Liu et al., 2010).

Logothetis et al. (2001) found that the root mean square value of LFP gamma vs. BOLD relationship is roughly linear. But when the LFP or BOLD is compared to the stimulus, the relationship is non-linear (Heeger and Ress, 2002). Huttunen et al. (2008) also found that the LFP/BOLD response, as a function of stimulus frequencies, is non-linear, but the LFP vs. BOLD relationship is quite linear. Devonshire et al. (2012) found that a non-linearity exists in sub-cortical regions but not in the cortex. Sanganahalli et al. (2009) found that both LFP and MUA show a linear relationship with hyperemic component [BOLD, cerebral blood volume (CBV), CBF] from the cortex in rats during forepaw stimulation with low frequency stimulation (1.5–3 Hz). The same group also showed that the relationship among LFP, MUA, and BOLD might be different in the cortex and sub-cortical regions in a recent study (Sanganahalli et al., 2016), but did not specifically quantify whether the relationship is linear. Magri et al. (2012) proposed to use mutual information to study the relationship between LFP band limited power and BOLD, which takes any non-linearity into account, however, they did not specifically measure how much non-linearity was present in the LFP-BOLD relationship.

To the best of our knowledge, there has not been a study specifically focusing on non-linearity in the relationship between spontaneous LFPs and BOLD in the cortex. So far, any non-linearities discovered in cortex seem to refer to the LFP vs. input stimulus, or BOLD vs. input stimulus relationship, but not the LFP and BOLD relationship. However, there have been some studies revealing the non-linearity between the BOLD signal and neural activity measured by methods other than LFP recordings (Devor et al., 2003; Jones et al., 2004; Sheth et al., 2004; Hewson-Stoate et al., 2005; Hoffmeyer et al., 2007; de Zwart et al., 2009; Liu et al., 2010). Since these studies suggests that there might be a non-linearity between BOLD and neural activity, it is worth trying to quantify if there is any non-linearity in the LFP-BOLD relationship. Please note that the linear relationship discussed in this paper refers to the simple y = kx + b relationship, and the aim of this paper is to discuss whether this holds true for LFP-BOLD relationship, and if not, how the Pearson correlation is affected.

In our study, we took advantage of the previously acquired data with simultaneous LFP and fMRI acquisition in rodents. Using data-driven approaches, we found a non-linear relationship existing between LFP power and BOLD under isoflurane (ISO) anesthesia but not under dexmedetomidine (DMED) anesthesia. This non-linearity seems to come from the non-Gaussian distribution of LFP power under ISO anesthesia. Subsequent studies show that ultimately, the non-linearity may come from the intrinsic properties in LFP power, and LFP amplitude might be more desirable if the non-linearity is a concern. Despite the existence of non-linearity, we also found that it is not usually substantial enough to influence traditional Pearson correlation-based analysis.

## Materials and Methods

### Simultaneous fMRI Imaging and LFP Recording

**Figure 1:**
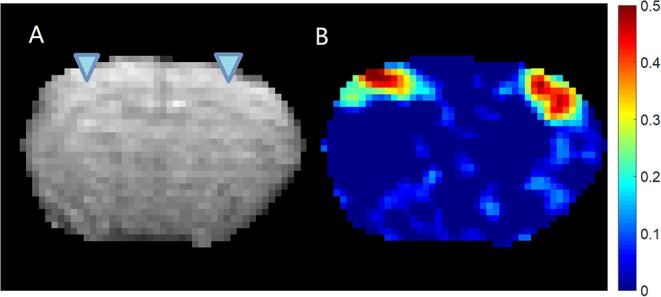
EPI image **(A)** and cross-correlation **(B)** between the LFP recorded from left S1 area and the BOLD signal. The location of the electrodes was indicated by the triangles overlaid on the EPI image. It can be seen that for this subject, the LFP recorded from left S1 area shows significant localized correlation with the BOLD signal near the electrodes on both hemispheres. The colormap for the correlation is shown on the right.

All animal experiments were performed in compliance with NIH guidelines and were approved by the Emory University Institutional Animal Care and Use Committee. Previously acquired data from 36 Sprague–Dawley rats (male, 200–300 g, Charles River) were used in this study. For each rat, first the surgery was performed to implant the glass electrodes in bilateral S1FL (primary somatosensory of forelimb) areas under 2% isoflurane (ISO) anesthesia (Figure 1 shows the EPI image of a typical subject with the locations of the electrodes, as well as the LFP vs. BOLD cross-correlation map). Then, simultaneous resting state-fMRI scans and LFP recordings were acquired, first under a variety of ISO concentrations ranging from 1.2 to 2% (ISO, 96 sessions), and later under dexmedetomidine (DMED, 219 sessions). For DMED studies, a bolus of 0.025 mg/kg dexmedetomidine was injected subcutaneously. Isoflurane was disconnected 10 min afterwards, and a continuous subcutaneous infusion of dexmedetomidine (0.05 mg/kg/h) began. The dose was increased by a factor of three (0.15 mg/kg/h) after ~1.5 h, following the protocol for prolonged sedation described in Pawela et al. (2009). The DMED scans were conducted >3 h after switching from ISO to avoid any residual ISO effects (Magnuson et al., 2014). Each session is 500 s long. A full description of the methods is given in previous publications (Pan et al., 2010, 2011). All physiological parameters were monitored and maintained within normal ranges, including rectal temperature, respiration rate, oxygen saturation and cardiac rate. The animals were euthanized at the end of the experiment.

Single slice gradient echo EPI scans were obtained on a 9.4T small animal MRI system (Bruker, Billerica, MA) with scan parameters: TR/TE = 500/15 ms, voxel size = 0.3 × 0.3 × 2 mm, matrix size = 64 × 64, FOV = 1.92 × 1.92 cm, number of repetitions = 1,000, number of dummy scans = 20. To improve the homogeneity of the magnetic field, the volume of interest (6 mm^3^) was shimmed using FASTMAP (Gruetter, 1993). Manual shimming adjustment was then applied when necessary to improve the field homogeneity of the selected slice. The imaging slice was set to the coronal plane that covers bilateral S1FL areas, where the glass recording electrode tips were implanted.

Because the whole dataset was acquired over a period of several years, there were two different sets of LFP recording parameters: (1) ×500 amplified, 0–100 Hz bandpass-filtered, 60 Hz notch-filtered, 12 kHz sampling rate, and ~10 min acquisition length (Pan et al., 2011), and (2) ×1,000 amplified, 0.1 Hz−5 kHz bandpass-filtered, 60 Hz notch-filtered, and 12 kHz sampling rate, and ~14 min acquisition length (Pan et al., 2013). However, these differences in the recording parameters are eliminated in the LFP pre-processing, where the LFP was band-pass filtered to 1–100 Hz, the amplitude was normalized so that the LFP broadband power (1–100 Hz) in each scan session has zero mean and unit variance, and the excessive LFP segments were truncated to match the length of fMRI data.

### LFP Data Pre-processing

The gradient switching that occurs during EPI acquisition induces voltage changes in the recorded LFP due to Faraday's law of induction. Such gradient-induced artifacts were removed following established methods, The denoised LFP signal was then low pass filtered to 100 Hz using to remove any residual artifacts, and down-sampled from 12 KHz to 500 Hz to reduce file size and computation cost. A 10 TR-long segment of raw LFP trace and the denoised LFP trace of a typical subject (same as the one shown in Figure 1) were shown in Figures S1A,B.

To obtain the LFP power time course, first a 1 s long sliding window was applied, then within the window, the power spectral density (PSD) function was estimated using Welch's method (four segments, 50% overlap). The PSD was integrated over a range of frequency bands (delta 1–4 Hz, theta 4–8 Hz, alpha 8–12 Hz, low frequency beta 12–25 Hz, high frequency beta 25–40 Hz, and gamma 40–100 Hz) to produce the LFP band-limited power (BLP) time courses. The sliding window has an overlap of 50%, meaning it moves 0.5 s at each step to match with the fMRI temporal resolution. The LFP BLP time courses were then band-pass filtered (0.01–0.1 Hz for ISO and 0.01–0.25 Hz for DMED). We chose these cut-off frequencies because a previous study (Pan et al., 2013) has demonstrated that frequencies below 0.1 Hz in ISO data or below 0.25 Hz in DMED data exhibited higher BOLD/BLP coherences, when compared to higher frequencies. Finally, the BLP time courses from the same scan were normalized by a common scaling factor, such that the standard deviation of the broadband power is equal to 1, which makes the datasets with various amplitudes comparable with each other.

### FMRI Data Pre-processing

First the fMRI data was corrected for motion using SPM 12. The motion-corrected image series were then spatially smoothed using a Gaussian kernel with a FWHM of 2.8 voxel (2.8 × 0.3 = 0.84 mm). Finally, global signal and linear drift regression, as well as band-pass filtering (0.01–0.1 Hz for ISO and 0.01–0.25 Hz for DMED) were performed voxel-wisely. All data processing was performed on Matlab 2018b (The MathWorks, Natick, MA). The data will be available upon request.

### ROI Selecting and Quality Assurance

As a quality assurance step, the cross-correlation map of LFP bandlimited power vs. BOLD is calculated at the lag when the correlation is expected to reach the maximum (4 s for ISO and 2.5 s for DMED). If there were high cross-correlations near the electrodes, the dataset was selected as high-quality dataset. Please note that the initial data pool (315 scan sessions) includes all saved data, including those with substantial noises, motions, and/or unstable physiological conditions. Most of them were not suitable for further study and were excluded. We have used several metrics to assess the quality of the data, including the noise in LFP and BOLD, the residue motion, image distortion, and function connectivity in bilateral S1 areas. All of the metrics were manually inspected and labeled as “good,” “fair” or poor (the guideline of how the metrics were labeled can be found in Table 1). Any scan must have at most one “fair” metric, in order to be selected as usable data. By these criteria, 82 scans under ISO and 160 scans under DMED were selected out of the initial 315-scan data pool. The correlation between LFP power and BOLD were inspected, and if a scan session has a LFP-BOLD correlation higher than 0.2 and the correlation map is well-localized to sites surrounding electrodes, it is selected as high-quality data for further analysis. By this criterion, 22 scans under DMED were selected out of 219 scans, and 32 scans under ISO selected out of 96 scans. A 3 × 3 ROI was manually chosen from the cross-correlation map, and centered at the electrodes, where the correlations are the highest. Finally, the BOLD signal averaged over the ROI was normalized so that the BOLD time course in each scan session has zero mean and unit variance, thus, the BOLD signal is comparable with other fMRI scans. The LFP broadband power time course and the BOLD signal within the ROI of a typical subject (same as the one shown in Figure 1) were shown in Figure S1C.

**Table 1:** Guidelines of data quality metrics.

	**Good**	**Fair**	**Poor**
**LFP**			
Number of gradient induced artifact	= 1,020^a^		≠ 1,020
Residual noise in LFP	No large spike in de-noised LFP		More than one large spike in de-noised LFP
**BOLD**			
Trajectory of center of mass^b^	abs(Δx) ≤ 0.05 pixel and abs(Δy) ≤ 0.05 pixel	abs(Δx) ≤ 0.1 pixel and abs(Δy) ≤ 0.1 pixel and either abs(Δx) ≥ 0.05 pixel or abs(Δy) ≥ 0.05 pixel	abs(Δx) ≥ 0.1 pixel or abs(Δy) ≥ 0.1 pixel
DVARS^c^	DVARS ≤ 0.5%	0.5% ≤ DVARS ≤ 1% and the number of small spikes is fewer than 5	Either DVARS ≥ 1% (large spikes) or the number of small spikes is more than 5
Image distortion	No noticeable image distortion or signal loss		Noticeable image distortion or signal loss
Function connectivity (correlation between bilateral S1 areas)	Correlation ≥ 0.3 and the correlation map is localized^d^ to somatosensory network	Correlation ≥ 0.15 and either correlation ≤ 0.3 or the correlation map is not completely localized	Correlation ≤ 0.15 or unlocalized correlation
LFP-BOLD correlation	Correlation ≥ 0.2 for both S1 areas and the correlation map is well-localized to sites surrounding electrodes	Correlation ≥ 0.1 and either correlation ≤ 0.2 or the correlation map is not completely localized	Correlation ≤ 0.1 or unlocalized correlation

a *Since there are 1,000 TRs and 20 dummy scans, the number of gradient artifacts identified should equal 1,020. If not, it usually indicates high noise level or missing segments. Also the correct identification of gradient artifacts is crucial to register the timing*.

b *Center of mass shows if there is any shifting in x-y plane*.

c *DVARS is calculated based on Power et al. (2012). It is also an indicator of motion*.

d *The reason why localized high correlation is needed as a metric is because some scan session exhibit high correlation over a huge area of the brain, which is not normal and may be attributed to motion. The threshold for the correlation is not a “hard” threshold, but rather it is roughly the range of correlation we observed in the scan sessions that show localized correlation map. It is intended to provide a general idea of the correlation in such dataset. So if the correlation between bilateral S1 areas ≥ 0.3, the correlation map is not necessarily localized. But on the other hand, if the correlation map is localized, most of the time the correlation in S1 areas we observed is roughly in the range that is larger than 0.3*.

## Results

### LFP Power vs. BOLD Relationship Shows Non-linearity Under ISO

**Figure 2:**
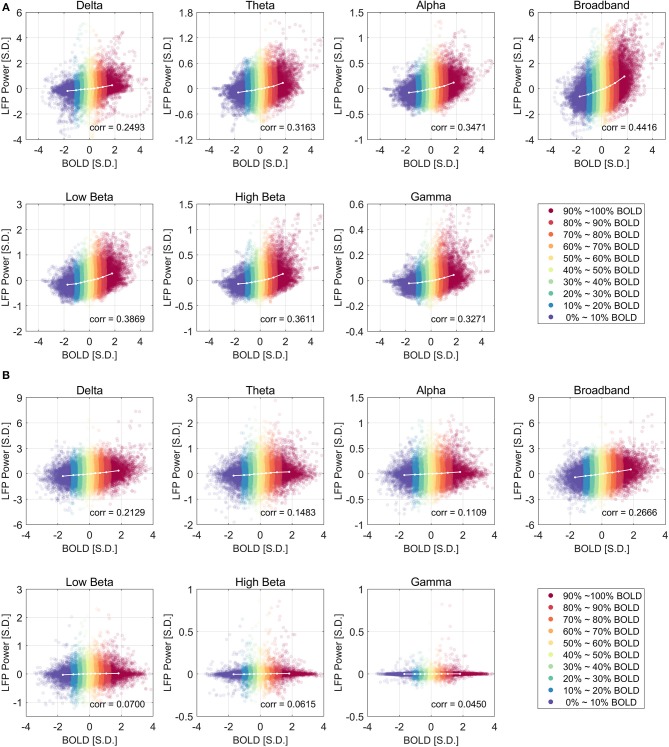
Scatter plot of LFP power vs. BOLD and the centroids of each category under ISO **(A)** and DMED **(B)**. The 10 BOLD categories are color-coded (Red for high BOLD value and Blue for low BOLD value). Each dot in the figure represents a time point with its BOLD value and LFP power value. In total there are 32,000 points under ISO, and 22,000 points under DMED, since each scan session has exact 1,000 time points. The cross-correlation between LFP power and BOLD is shown for each LFP band. The BOLD value and the LFP power value were expressed in units of standard deviation [S.D.]. Both BOLD and LFP power were normalized so that the standard deviation of BOLD is 1, and the standard deviation of LFP broadband power is also 1. For the individual LFP frequency bands, the summation of the power in the six bands at any given time points equals to the LFP broadband power (so any individual band will have a standard deviation lower than 1 standard deviation of LFP broadband power. Note that the display scale of different frequency bands may be different.

Figure 2 shows the relationship between LFP power and BOLD using a scatter plot. The x-axis shows the BOLD value measured in units of standard deviation of BOLD and the y-axis shows the LFP band limited power (BLP) values measured in units of standard deviation of LFP broadband power, at a lead of 4 s under ISO and a lead of 2.5 s under DMED.

Besides the scatter plot, there is also a line plot showing the mean LFP power vs. spontaneous BOLD relationship. It is obtained by evenly dividing the data into 10 groups based on the BOLD values, and then calculating the average LFP power within each BOLD group. Since this curve is based on the actual bivariate relationship, we call it “experimental LFP vs. spontaneous BOLD relationship.” It is evident that the LFP vs. spontaneous BOLD relationship is non-linear under ISO (panel A), whereas under DMED it is almost linear (panel B). In addition, while the scale of each plot is different (because the energy in each frequency band is different), the degree of non-linearity under ISO appears very consistent, suggesting that an underlying mechanism independent of the frequency bands induces the non-linearity. The degree of linearity under DMED also appears very consistent, except in high frequency bands, especially in gamma band. The correlation between gamma band under DMED and BOLD is only 0.0450, suggesting that they are only very weakly correlated. The reason why the correlation between high frequency band and BOLD under DMED is so small compared to the other frequency bands, is because the “signal-to-noise ratios” in these bands are small (The “signal” is the change in BOLD that is caused by LFP power changes, whereas the “noise” is the change in BOLD attributed to random fluctuations), which makes the LFP vs. spontaneous BOLD curve so flat. Under DMED anesthesia, there is little energy in these high frequency bands, whereas under ISO anesthesia, the energy in these bands is considerably higher. We have included the energy distribution of LFP power in the Figure S2.

**Figure 3:**
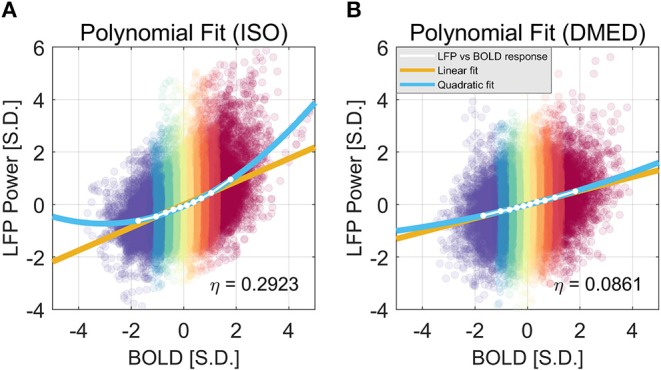
Quadratic Fitting of LFP power vs. BOLD response under ISO **(A)** and DMED **(B)**. The quadratic fitting captures the shape of the LFP power vs. BOLD response well. Under ISO the LFP power-BOLD relationship is much more non-linear than under DMED. Under ISO, the fitted coefficients a, b, c, are 0.0713, 0.4344, −0.0696, respectively. The *p*-values are 2.06e-05, 2.13e-10, 0.00029, respectively. The 95% confidence intervals are [0.0546, 0.0881], [0.4151, 0.4536], [−0.0944, −0.0449], respectively. Under DMED, the fitted coefficients a, b, c, are 0.0124, 0.2606, −0.0130, respectively. The *p*-values are 0.0122, 8.06e-11, 0.0465, respectively. The 95% confidence intervals are [0.0036, 0.0211], [0.2505, 0.2706], [–0.0258, −0.0003], respectively. Both ISO and DMED have a second order coefficient still significantly different from zero (*p* = 2.06e-05 < 0.05 under ISO, *p* = 0.0122 < 0.05 under DMED), however under ISO the magnitude of the coefficient is much larger, which is why we can visually see a curvature. The non-linearity, measured by the ratio between the second order term and the first order term, is also shown in the figure.

We also found that this non-linearity can be well-modeled by a simple second order polynomial function, shown in Figure 3. Let *x*_*i*_ and *y*_*i, j*_ be the averaged BOLD and LFP power of the *i*-th percentile group (*i* = 1, 2, … 10) in the *j*-th frequency band (*j* = 1, 2, … 6, 7, representing delta, theta, alpha, low-frequency beta, high-frequency beta, gamma, and broadband, respectively). The collection of [*x*_*i*_, *y*_*i, j*_] corresponds to the line plot in Figure 2. It appears that the non-linear relationship can be modeled as a quadratic function. To test that, we can predict the BOLD value ${\hat{y}}_{i,\;j}^{1}$ using the polynomials of the LFP power *x*_*i*_:

(1) $$\begin{array}{l} {\hat{y}}_{i,\;j}^{1}=f\left(x_{i}\right)=a_{j}{x_{i}}^{2}+b_{j}x_{i}+c_{j}=\beta_{j}^{T}X_{i}, \end{array}$$

where $\beta_{j}={\left[a_{j},\;b_{j},c_{j}\right]}^{T}$ and $X_{i}={\left[{x_{i}}^{2},\;x_{i},\;1\right]}^{T}$. We included a superscript in ${\hat{y}}_{i,\;j}^{1}$ because, in next section, we will introduce another model to predict the BOLD value. For each frequency band j, an optimal parameter set β_*j*_ can be found using the least squares method, such that the sum of squared error between the predicted BOLD and the actual BOLD is minimized:

(2) $$\begin{array}{lll} \beta_{j} & = & \underset{\beta_{j}}{\text{argmin}}\sum_{i=1}^{10}{\left(\;{\hat{y}}_{i,\;j}^{1}-y_{i,j}\right)}^{2}\\ \; & = & \underset{\beta_{j}}{\text{argmin}}\sum_{i=1}^{10}{\left(a_{j}{x_{i}}^{2}+b_{j}x_{i}+c_{j}-y_{i,j}\right)}^{2} \end{array}$$

The estimated parameters for the broadband power, their *p*-values, and 95% confidence interval can be found in the caption of Figure 3. The goodness of the fit can be measured by the root-mean-square-error (RMSE), and *R*^2^. Under ISO, the RMSE and *R*^2^ of the linear model are 0.0927 and 0.9639; the RMSE and *R*^2^ of the quadratic model are 0.0252 and 0.9977. It is evident that the quadratic model has a smaller RMSE and higher *R*^2^. Given that the second order coefficient also has a 95% confidence interval not overlapping with zero (0.0546, 0.0881), and the *p*-value is very small (2.06e-05), it can be concluded that there is substantial non-linearity in the LFP-BOLD relationship under ISO, and the quadratic model is more precise than the linear model. To quantitatively measure the non-linearity, naturally we would use the second order term, but the value of the second order term is also determined by the overall scale of the function. To normalize this effect, we propose to use the ratio of the second order term to the first order term as a measurement of how non-linear the function is. Recall Equation (1):

(3) $$\begin{array}{l} {\hat{y}}_{j}^{1}=f\left(x\right)=a_{j}x^{2}+b_{j}x+c_{j} \end{array}$$

To measure the non-linearity, we picked two points: the furthest point along the positive x-axis (denoted by [*x*_1_, *y*_1_]), and the origin (denoted by [*x*_0_, *y*_0_]). Taking the Taylor expansion at the origin, we have

(4) $$\begin{array}{l} f\left(x\right)=f\left(x_{0}\right)+f^{\prime }\left(x_{0}\right)\left(x-x_{0}\right)+\frac{f^{\prime \prime }\left(x_{0}\right)}{2}{\left(x-x_{0}\right)}^{2} \end{array}$$

The change along y-axis Δ*y* = *y*_1_ − *y*_0_ is determined by the change along x-axis Δ*x* = *x*_1_ − *x*_0_ (note that *x*_0_ = 0):

(5) $$\begin{array}{lll} \Delta y & = & f\left(x_{1}\right)-f\left(x_{0}\right)\;\\ \; & = & f^{\prime }\left(x_{0}\right)\left(x_{1}-x_{0}\right)+\frac{f^{\prime \prime }\left(x_{0}\right)}{2}{\left(x_{1}-x_{0}\right)}^{2}\;\\ \; & = & \left(2a_{j}x_{0}+b_{j}\right)\Delta x+\frac{2a_{j}}{2}\Delta x^{2}\;=a_{j}\Delta x^{2}+b_{j}\Delta x, \end{array}$$

which consists of the first order term and the second order term. Since the BOLD distribution is approximately Gaussian, if we assess the non-linearity using the furthest point along the positive x-axis, which is the group mean of 90–100% BOLD, then Δ*x* would be 1.819. The non-linearity in the LFP—BOLD relationship in the j-th frequency band, denoted as η_*j*_, can be calculated using the following equation:

(6) $$\begin{array}{l} \eta_{j}=\frac{2nd\;order\;term}{1st\;order\;term}=\frac{a_{j}\Delta x^{2}}{b_{j}\Delta x} \end{array}$$

Using this formula, the non-linearity in LFP broadband power vs. BOLD relationship is 0.2923 and 0.0862 under ISO and DMED, respectively.

### The Non-linearity Might Be Induced by the Non-gaussian Distribution of LFP Power

**Figure 4:**
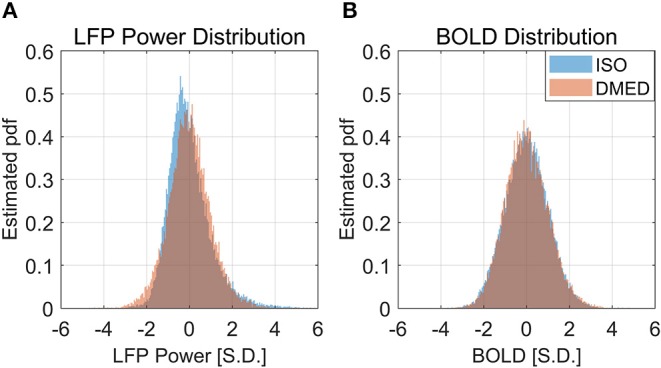
Histogram of LFP broadband power, BOLD under ISO **(A)** and DMED **(B)**. It can be seen that the LFP power under ISO is non-Gaussian distributed.

It is also worth noting that in Figure 2, the scatter plot reaches further along the positive y-axis, especially for the 90–100% BOLD group, which makes the LFP vs. spontaneous BOLD relationship appear as a curve. Therefore, the asymmetry along y-axis, or in another word, the skewed distribution of LFP power, might be the reason why the LFP—BOLD relationship is non-linear. Figure 4 shows the distributions of LFP power and BOLD, where it can be seen that the LFP power under ISO is right tailed. This can be quantitatively confirmed by the skewness (a skewness >1 is often considered as highly skewed). Together with a kurtosis of 5.3575, which is much higher than 3 (Gaussian distributions always have a kurtosis of 3), it can be concluded that the LFP power under ISO is substantially non-Gaussian.

The skewness and kurtosis were calculated using the following equations:

(7) $$\begin{array}{l} Skew\left[X\right]=E\left[{\left(\frac{X-\mu }{\sigma }\right)}^{3}\right], \end{array}$$

(8) $$\begin{array}{l} Kurt\left[X\right]=E\left[{\left(\frac{X-\mu }{\sigma }\right)}^{4}\right], \end{array}$$

where *X* is the random variable, μ is the mean value of *X*, σ is the standard deviation. The skewness and kurtosis of the distributions were shown in Table 2.

**Table 2:** Skewness and kurtosis of LFP power and BOLD under ISO and DMED anesthesia.

	**LFP ISO**	**LFP DMED**	**BOLD ISO**	**BOLD DMED**
Skewness	**1.0473**	0.2235	0.1191	0.1802
Kurtosis	**5.3575**	**4.1773**	3.0987	3.1723

*The skewness or kurtosis that deviates from a Gaussian distribution were highlighted in bold*.

The LFP power under DMED also has a kurtosis larger than 3, but it is relatively Gaussian when compared to LFP power under ISO. The BOLD signal, on the other hand, is approximately Gaussian-distributed, regardless of the anesthetizing agent, because the skewness is near 0 and the kurtosis is near 3.

It is apparent from the histogram, as well as from the skewness and kurtosis that the LFP power under ISO is the most non-Gaussian signal. Given the fact that the LFP power vs. spontaneous BOLD relationship under ISO is also the one showing substantial non-linearity, we hypothesized that these two findings are linked together, and the non-linearity may come from the non-Gaussian distribution of LFP power.

### Least Square Fitting of LFP vs. Spontaneous BOLD Relationship

To validate this hypothesis, we propose to obtain a theoretical LFP power vs. spontaneous BOLD relationship by making some assumptions, and then determine if the experimental curve matches the theoretical one.

A natural assumption would be that a positive spontaneous BOLD event is evoked by higher than average LFP power, and negative spontaneous BOLD event is evoked by lower than average LFP power. If there was a purely deterministic relationship between the two, we would see 90% percentile BOLD corresponds to 90% percentile LFP power. The relationship is stochastic, so the final observation is contaminated by random noise. But the mean value of BOLD within a certain percentile range should still correspond to the mean value of LFP power within the same percentile range even in the presence of noise. For example, the mean effect of LFP power with 90–100% percentile value should, on average, evoke a spontaneous BOLD event with 90–100% percentile value. The assumption made here is weaker than the assumption of linearity. In a special case where both LFP and BOLD follow Gaussian distributions, it is equivalent to the linear assumption; but in general, if any of the distributions are non-Gaussian, such an assumption should still faithfully reflect the averaged relationship between LFP and BOLD.

**Figure 5:**
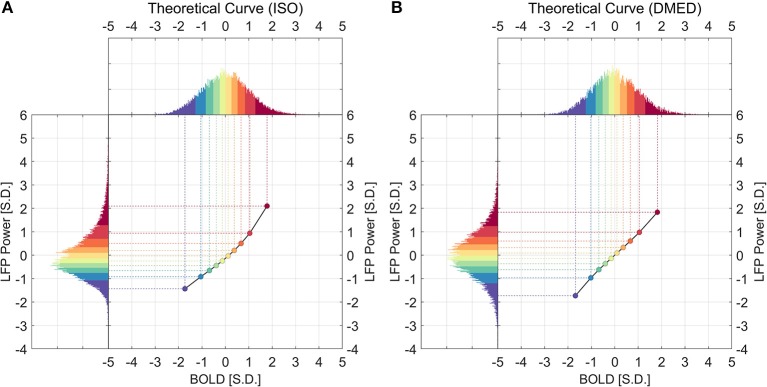
Histograms of LFP, BOLD, and the derived theoretical LFP vs. BOLD response under ISO **(A)** and DMED **(B)**. For any given point in the LFP vs. BOLD response, the x-axis shows the averaged BOLD value of the BOLD group, while the y-axis shows the averaged LFP broadband power value of the corresponding LFP group.

Figure 5 illustrates how the theoretical LFP vs. spontaneous BOLD relationship was obtained. First, the distributions of both LFP and BOLD were evenly divided into 10 groups, which is shown in the color-coded histograms. Next, the mean LFP power in a percentile group (y-axis value) was mapped to the mean BOLD in the corresponding percentile group (x-axis value). It is worth noting that the theoretical curve solely depends on the overall distributions of LFP power and BOLD, whereas the experimental curve was obtained from the one-to-one relationship in the data points.

Since the LFP-BOLD relationship seems consistent across all frequency bands, for the sake of robustness, we used the LFP broadband power to derive a single theoretical LFP vs. spontaneous BOLD curve. A least square fitting was then performed to find the optimal scaling factor for each frequency band, which minimizes the summed squared difference between the scaled theoretical curve and the experimental curve. Let ${\tilde{x}}_{i}$ and ỹ_*i, j*_ be the BOLD and LFP broadband power of the *i*-th point in the *j*-th frequency band in the theoretical curve (Figure 5), respectively. Since it is hypothesized that the non-linearity comes from the non-Gaussian distributions, which have been taken into account in the theoretical curve, the predicted LFP-BOLD relationship $\left[{\hat{x}}_{i},\;{\hat{y}}_{i,\;j}^{2}\right]$ in each frequency band is then a scaled version of the theoretical curve in the LFP broadband power:

(9) $$\begin{array}{l} {}[{\hat{x}}_{i},\;{\hat{y}}_{i,\;j}^{2}]=[{\tilde{x}}_{i},\;\theta_{j}{\tilde{y}}_{i,7}], \end{array}$$

where θ_*j*_ is the scaling factor for *jth* frequency band. Note that $x_{i}={\tilde{x}}_{i}=\;{\hat{x}}_{i}$ (because they all represent the averaged BOLD within the [10 × (*i* − 1)%, 10 × *i%*] percentile group). The optimal scaling factor θ_*j*_ was found such that the sum of squared error between the predicted curve and the experimental curve is minimized:

(10) $$\begin{array}{lll} \theta_{j} & = & \underset{\theta_{j}}{\text{argmin}}\sum_{i=1}^{10}{\left(\;{\hat{y}}_{i,\;j}^{2}-y_{i,j}\right)}^{2}\\ \; & = & \underset{\theta_{j}}{\text{argmin}}\sum_{i=1}^{10}{\left(\theta_{j}{\tilde{y}}_{i,7}-y_{i,j}\right)}^{2} \end{array}$$

**Figure 6:**
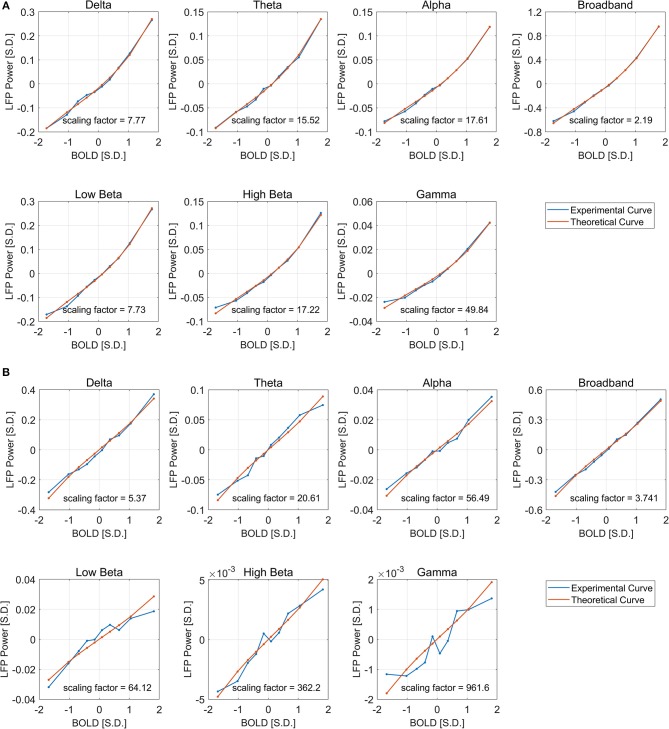
Least square fitting of experimental and theoretical LFP vs. BOLD responses under ISO **(A)** and DMED **(B)**. The fitting is good across all frequency bands and both anesthesia conditions. The scaling factor shows the amount of amplification needed by the experimental response to match with the theoretical one.

It can be seen from Figure 6 that the derived theoretical LFP vs. spontaneous BOLD curve match fairly well with the experimental ones, suggesting that the non-linearity may come from the non-Gaussian distribution of LFP power, although the experimental curves do deviate from the theoretical ones in low frequency beta, high frequency beta, and gamma bands under DMED. The reason again is the “Signal-to-noise” ratios mentioned earlier are very small in these bands, making them very sensitive to random noise, or fluctuations in BOLD that is not caused by LFP changes. We also increased the number of groups from 10 to 40 to get the fitting in finer grid. The fitting results (Figure S3) were consistent with the one obtained using 10 groups.

### The Ultimate Source of Non-linearity

We have shown that the LFP power vs. BOLD relationship is non-linear, and that such non-linearity may come from the non-Gaussian distribution of LFP power. But there are still a few questions remaining. (1) What exactly makes LFP power under ISO non-Gaussian? (2) Why can the linearity be modeled by a simple second order polynomial fit? (3) And why does this non-linearity seem to exist only under ISO anesthesia?

We hypothesized that the ultimate reason for the non-linearity is that taking the power of LFP induces a second order non-linearity. So alternatively, we can look at the LFP amplitude—BOLD relationship. [The “amplitude” we use here is simply the square root of the LFP power. For a narrow band like alpha band, gamma band, this is closer to the amplitude of the signal, whereas for the broadband signal (1–100 Hz), it is more like a root-mean-square (r.m.s.) values in a 1 s time window]. The reasoning for the hypothesis is the following.

**Figure 7:**
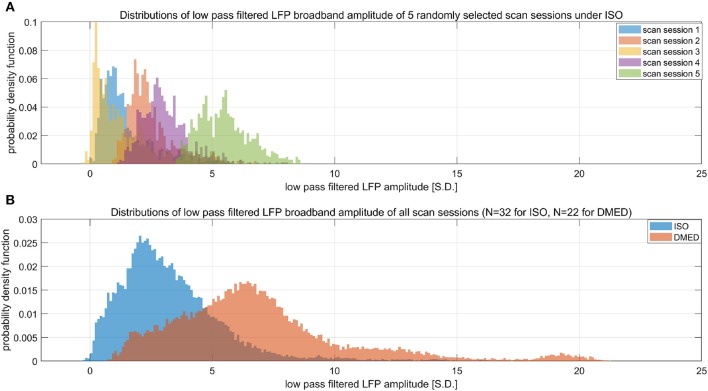
Histograms of low pass filtered LFP broadband amplitude. **(A)** Shows five randomly selected scan sessions under ISO to illustrate the variety in their mean value. **(B)** Shows the overall distribution of low pass filtered (under 0.1 Hz under ISO and under 0.25 HZ under DMED) LFP broadband amplitude obtained from the entire dataset (*N* = 32 for ISO, *N* = 22 for DMED). Since low pass filtering preserves the direct current component, it can be seen that the LFP amplitude under ISO actually has a much lower baseline (mean value) when compared to under DMED. The unit in the figure is 1 standard deviation (S.D.) of the band pass filtered (0.01–0.1 Hz under ISO and 0.01–0.25 Hz under DMED) LFP broadband amplitude. So the scale of the signal is the same as the ones shown in previous figures, with the only difference being the superposition of the direct current component preserved by switching band pass filtering to low pass filtering.

**Figure 8:**
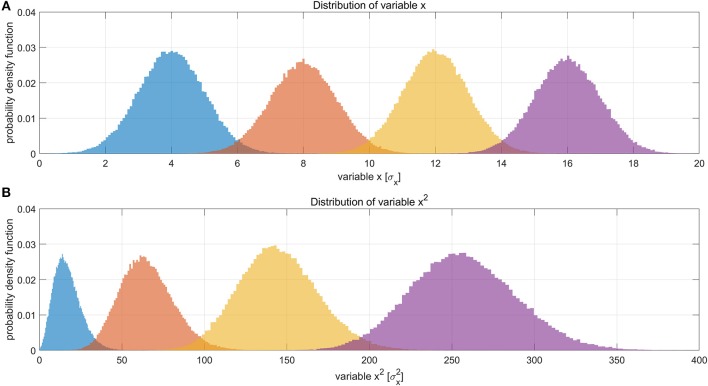
Illustration of how Gaussian distributions can transform to non-Gaussian ones by taking the power of two, and the degree of non-linearity is influenced by the mean value (baseline) of the original distribution. **(A)** Shows four different distributions of a hypothetical variable x, representing the LFP amplitude. The histograms were obtained by Monte Carlo simulation of 100,000 points for each distribution. The four distributions have the same standard deviation (σ = 1σ_*x*_) but different mean values (μ = 4σ_*x*_, 8σ_*x*_, 12σ_*x*_, 16σ_*x*_, respectively). **(B)** Shows the distributions of variable *x*^2^. The unit in panel B is $\sigma_{x}^{2}$. It can be clearly seen that the one with the lowest mean value (blue), become much more non-Gaussian after taking the power of two, whereas the one with the highest mean value (purple) still remains approximately Gaussian. This suggests that the non-Gaussian distribution of LFP power under ISO (shown in Figure 4) may partly come from taking the power of two.

Suppose LFP amplitude follows a Gaussian distribution, so the LFP amplitude—BOLD relationship is linear. The LFP power is the square of LFP amplitude, which transforms the original Gaussian distribution into a non-Gaussian one and, as a consequence, makes the LFP power—BOLD relationship become a quadratic curve. Since the LFP amplitude–BOLD relationship is assumed to be linear, any non-linearity observed in LFP power—BOLD relationship is equivalent to the non-linearity in LFP power–LFP amplitude relationship. For a fixed curve, like y = x^2^ in the LFP amplitude—LFP power relationship, the non-linearity depends on the baseline (mean value) of the signal. In the case where the LFP amplitude has a very high baseline, like the one under DMED anesthesia, the non-linearity η, which is the ratio of the second order change to the first order change, become relatively small, while in the case where the LFP amplitude has a very low baseline, like the one under ISO anesthesia, the non-linearity η becomes relatively large. To illustrate this effect, we applied low pass filtering (0.1 Hz under ISO and 0.25 Hz under DMED) instead of band pass filtering, so that the direct current (DC) component, or the mean value of the signal can be preserved. Each scan sessions were again normalized using the same scaling factor from the band pass filtered signal, so that the alternating current (AC) component (0.01–0.1 Hz under ISO and 0.01–0.25 Hz under DMED) of the broadband signal (1–100 Hz) has a standard deviation of 1. Figure 7 shows the distribution of the low pass filtered LFP broadband (1–100 Hz) amplitude time courses (with panel A shows five randomly selected scan sessions under ISO, and panel B shows the overall distributions under ISO and DMED). It is evident that the LFP amplitude has a significantly higher baseline under DMED when compared to under ISO. Figure 8 shows the Monte Carlo simulation of how hypothetically four Gaussian distributions with different mean values (representing the LFP amplitude with different baseline) will transform into non-Gaussian ones by taking the power of two. The range of the mean values were selected to cover the distributions of LFP amplitude. It can be seen that for the one with the lowest mean value (blue), the transformed distribution clearly became non-Gaussian (to be more specific, positively skewed or right-tailed, just like the distribution of LFP power under ISO), whereas the one with the highest mean value (purple) still remains approximately Gaussian after taking the power of two. These suggest that the non-Gaussian distribution may come from taking the power of two, and since under ISO the baseline is lower, the non-linearity becomes greatly amplified. The hypothesis that the non-linearity comes from the nature of power might answer all of the questions at the same time, so it is very worthwhile to test whether this hypothesis is true or not.

**Figure 9:**
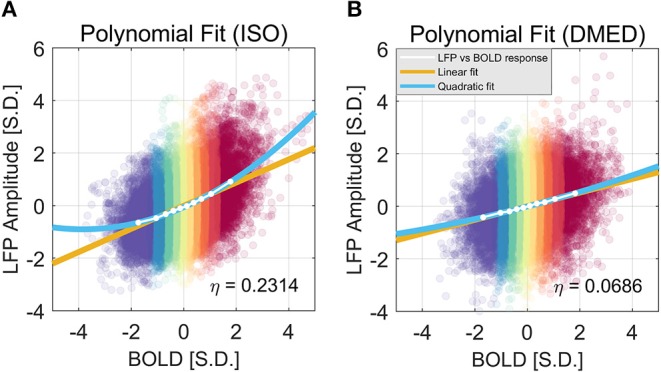
Quadratic Fitting of LFP amplitude vs. BOLD response under ISO **(A)** and DMED **(B)**. It can be seen that, under ISO, the non-linearity in LFP amplitude–BOLD relationship is smaller than the one in LFP power—BOLD relationship, although the remaining non-linearity is still considerably larger than the one under DMED. Under ISO, the fitted coefficients a, b, c, are, 0.0570, 0.4386, −0.0606, respectively. The *p*-values are, 7.24e-6, 1.39e-11, 6.35e-5, respectively. The 95% confidence intervals are [0.0456, 0.0685], [0.4255, 0.4518], [−0.0775, −0.0437], respectively. Under DMED, the fitted coefficients a, b, c, are 0.0098, 0.2584, −0.0099, respectively. The *p*-values are 0.0321, 8.09e-11, 0.1085, respectively. The 95% confidence intervals are [0.0011, 0.0184], [0.2484, 0.2684], [−0.0226, 0.0028], respectively.

**Figure 10:**
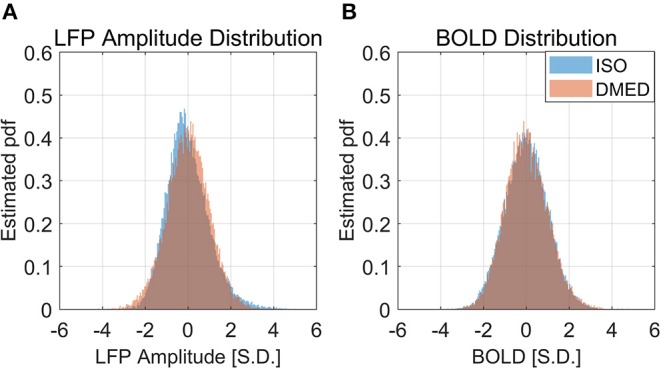
Histogram of LFP broadband amplitude, BOLD under ISO **(A)** and DMED **(B)**. It can be seen that the LFP amplitude under ISO is less skewed than LFP power, but is still non-Gaussian distributed.

Figure 9 shows the LFP amplitude vs. BOLD scatter plot. The settings are identical to Figure 3, except the LFP amplitude is substituted for LFP power. The histogram of LFP amplitude and BOLD are shown in Figure 10. While it appears that the LFP amplitude-BOLD relationship is still non-linear and the LFP amplitude is non-Gaussian, the non-linearity, measured by the ratio of second order term to first order term, does show a decrease when switched from LFP power to LFP amplitude (from 0.2923 to 0.2314). Table 3 also shows that, the skewness of LFP amplitude is closer to 0 compared to LFP power. The kurtosis also became smaller, thereby the LFP amplitude is more Gaussian than LFP power. Using LFP amplitude does make the LFP–BOLD relationship a little bit more linear, although there are still other unknown factors that account for the remaining non-linearity.

**Table 3:** Skewness and kurtosis of LFP amplitude and BOLD under ISO and DMED anesthesia.

	**LFP ISO**	**LFP DMED**	**BOLD ISO**	**BOLD DMED**
Skewness	**0.5870**	0.0170	0.0920	0.1753
Kurtosis	**3.8742**	3.4888	3.0895	3.0853

*The skewness or kurtosis that deviates from a Gaussian distribution were highlighted in bold*.

### The Non-linearity Does Not Greatly Influence Pearson Correlation

**Figure 11:**
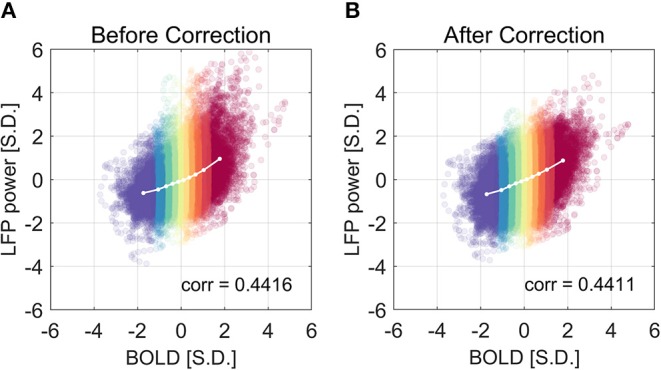
The correlation coefficient is not improved after the non-linear correction. The correlation coefficient before and after non-linearity correction was shown in **(A)** and **(B)**, respectively. The experimental LFP power vs. BOLD relationship is more linear after the non-linear correction, but there is little change in the correlation coefficient.

We have shown that the LFP power–BOLD relationship is non-linear, and such non-linearity may come from the non-Gaussian distribution of LFP power. A further question is how this non-linearity will influence the data analysis, namely the correlation between LFP and BOLD. Theoretically, Pearson correlation coefficient only measures linear dependency. In the case that the relationship is extremely non-linear, more generalized analysis methods that do not assume linear relationship (e.g., mutual information) are desirable. We corrected the non-linearity of the LFP power—BOLD data by mapping the LFP-power distribution back to a Gaussian distribution using the inverse of the theoretical LFP-BOLD curve. From Figure 11, we can see that the non-linearity is reduced, judging by the non-linearity metric defined by Equation (6). However, the Pearson correlation is not significantly changed [the mean value of the Pearson correlation before and after correction were 0.4416 and 0.4411, respectively. The 95% confidence intervals of the Pearson correlation before and after correction were (0.4327, 0.4505), (0.4321, 0.4500), respectively].

## Discussions

### LFP—BOLD Relationship Can Be Non-linear

Simultaneous LFP and fMRI data acquisition is an essential tool for understanding the connection between neural activity and the BOLD signal contrast. So far there is not a lot of detailed discussion about the non-linearity between LFP and BOLD recorded in the cortex. Logothetis et al. (2001) first described the relationship between LFP amplitude (more precisely, the root-mean-square value of gamma band LFP time course) and BOLD as roughly linear. However, later in a review paper (Heeger and Ress, 2002), it was stated that their relationship is monotonic but non-linear, because a 12% stimulus contrast evoked about half the maximum fMRI response, but much less than half the maximum LFP and MUA (this does not actually contradict what Logothetis et al. found, because the non-linearity here is in the LFP/BOLD as a response to the stimulus, whereas the LFP directly plotted against BOLD is still roughly linear). Huttunen et al. (2008) performed a simultaneous LFP and BOLD recording under controlled fore paw stimulus with different frequencies. It is worth mentioning that, although many papers cited (Huttunen et al., 2008) as one revealing non-linearity in neural–hemodynamic coupling, the non-linearity was in the BOLD/LFP response as a function of stimulus frequencies. In terms of the LFP-BOLD relationship, which is the main focus of our paper, they discovered a strikingly high Pearson correlation between LFP and BOLD (*r* = 0.97 under urethane and *r* = 0.89 under alpha-chloralose). This correlation-based analysis suggests that the relationship between LFP and BOLD is actually quite linear under these anesthesia conditions. (Magri et al., 2012) proposed to use mutual information to study the relationship between LFP band limited power and BOLD in resting-state. Mutual information is the most general measure of the statistical dependency, and thus takes into account any non-linearity, which is superior to Pearson correlation in the presence of considerable non-linearity. However, they did not specifically measure how much non-linearity is present in the LFP-BOLD relationship. Devonshire et al. (2012) have found a non-linear relationship between the LFP responses and the BOLD responses (summed in a 40 s time window after stimulus) in sub-cortical regions, which manifests itself in a power law curve. In the meantime, they also found that the LFP-BOLD relationship was linear in S1 region. In our work, we proposed a method to quantify the non-linearity that is tailored for extremely noisy data like LFP and BOLD. The correlation between LFP and BOLD is 0.441 ± 0.124 under ISO, *n* = 32, and 0.267 ± 0.115 under DMED, *n* = 22. Given this range of correlation, it is not possible to provide a deterministic prediction for one variable if the other is known. By dividing the data into several subgroups based on BOLD values, and then averaging the LFP power within each BOLD subgroup, we obtained a LFP—BOLD relationship in which a non-linearity can be visually observed under ISO anesthesia. The group average is more robust to the randomness in the data, which enables the quantification of non-linearity.

From the 32 scan sessions under ISO, we observed a substantial non-linearity independent of the frequency band, in the form of second order polynomial fit. The consistency here suggests that the curved shape response is not a coincidence, but an actual phenomenon that is hiding under the noisy LFP—BOLD data. In contrast, the relationship is found to be linear in the 22 scan sessions under DMED, which suggests that such non-linearity is subject to the type of anesthesia. Isoflurane is commonly used to induce anesthesia, perform surgical procedures, and maintain a deep level of unconsciousness in rodents during setup for fMRI. At high isoflurane doses (>1.8%), widespread cortical neural burst suppression (Rehberg et al., 1996) results in reduced cortical excitation and reduced spatial sensitivity of functional connectivity, therefore, anesthesia is typically switched to an agent that is less suppressive of neural activity for during fMRI acquisition. However, at lower dosages (<1.5%), functional activity and connectivity remain fairly intact so there have been some studies using isoflurane during imaging as well (Guilfoyle et al., 2013; Kalthoff et al., 2013; Liu et al., 2013). In addition to the burst-suppression, isoflurane is also a vasodilator, which affects the cerebral blood flow (CBF), and thus will affect the BOLD signal. On the other hand, dexmedetomidine is less suppressive to neural activity, and induces a neural state very similar to natural sleep, while simultaneously causing muscular relaxation (Nelson et al., 2003). Dexmedetomidine is therefore more preferable in functional MRI in terms of the alterations of neural activity. However, dexmedetomidine is a vasoconstrictor, which also affects the CBF. We believe that comparing the data obtained under ISO and DMED can provide results that are more generalizable than using only one anesthetic agent. Thus, far, many studies performed with different anesthetic agents seem to conclude different frequency bands in LFP that best correlate with BOLD. From the ISO and DMED data presented here, it is possible that the apparent discrepancy is caused by the energy distribution under the specific anesthetic state. For example, the delta band might best correlate with BOLD if the anesthesia shifts the energy toward lower frequency bands. Further studies need to be performed on other anesthetic agents to support this hypothesis.

### Non-linearity in LFP vs. BOLD Under ISO Anesthesia May Reflect the Non-gaussian Distribution of LFP Power

We proposed that the non-linearity may come from the highly skewed, non-Gaussian distribution of LFP power. We derived a theoretical LFP—BOLD curve solely from the overall distributions of LFP power and BOLD, without knowing any LFP-BOLD dynamics for any specific data points. The goodness of the fit over all the frequency bands under both ISO and DMED suggests that the non-linearity in the LFP vs. BOLD relationship could come from the non-Gaussian distribution of LFP power.

Ultimately, the non-linearity may come from the LFP power itself, which has an intrinsic non-linear property when compared to the LFP amplitude. It is still questionable whether the LFP amplitude itself is linear vs. BOLD, and the observed non-linearity in LFP power may not come solely from taking the square. Nevertheless, the skewness, kurtosis, and the non-linearity measured by the second order/first order ratio are all smaller in LFP amplitude than in LFP power, suggesting the former one is relatively more linear. In addition, such hypothesis theoretically answered the three questions at the same time, making it quite reasonable, although it is still not fully confirmed. It appears that the non-linearity depends on the type of anesthesia, given the fact that in most studies the LFP—BOLD relationship is considered to be linear with the use of many different anesthetic agents, the non-linearity observed here might originated from something specific under ISO. One possible explanation is the burst-suppression, because the constant switching from “on” and “off” states, combined with a smoothing effect from the low pass filtering, does seem quite non-linear, and it is worth investigating in the future.

### Implication for Future Studies

First, this study confirms that under some specific conditions, the LFP—BOLD relationship during spontaneous activity can be non-linear. Therefore, caution should be taken whenever analyzing simultaneous LFP and fMRI data, because apparently, depending on the animal model and the anesthetic agent used, there might be unexpected non-linearity present in the data. Mathematically, it is not accurate to use the Pearson correlation coefficient to describe the dependency between two variables when one variable is Gaussian distributed and the other is not Gaussian distributed (or the dependence is non-linear). The extent to which the accuracy is compromised depends on how much the distribution deviates from Gaussian distribution.

Secondly, we have evidence supporting the idea that the intrinsic properties of LFP power might contribute to some of the non-linearity between LFP power and BOLD. It is worth noting that, the LFP power is widely used because most studies involve the band limited LFP power in some specific frequency bands. Since the integration over a frequency band yield the LFP power in that band, naturally the LFP power would become the first option. If the LFP power does induce non-linearity, it might be worthwhile to think twice about whether to use LFP power or LFP amplitude, or at least to check the linearity when using LFP power. Currently, the most common ways to get LFP amplitude are wavelet transform or Fourier transform, Hilbert transformation, and direct band pass filtering. Other than the one obtained from Fourier transform, the different LFP amplitude components in different frequency bands are not orthogonal, and it is somewhat difficult to get back to the original form of signal.

Despite the fact that the LFP power—BOLD relationship is substantially non-linear, the correction of non-linearity between the two only slightly changes the correlation coefficient (from 0.4416 to 0.4411, not statistically significant). This leads to the conclusion that, in the presence of substantial non-linearity in this specific situation under ISO, the Pearson correlation coefficient is still a valid measurement of the dependency between LFP and BOLD, and in other cases where non-linearity is usually not detectable, Pearson correlation is a reasonable metric.

### Technical Limitations

It is worth mentioning that the dataset for LFP—BOLD relationship analysis was deliberately chosen to have high cross-correlation between LFP power and BOLD around S1FL areas. While this ensures the overall quality of data, it may induce some bias as well. Only a very small portion of the dataset is usable for the analysis (32 scans out of 96 scans under ISO, and 22 scans out of 219 scans under DMED). Right now, the reason why the correlation coefficient can vary drastically in adjacent scans in the same rat, even with almost identical physiological conditions, is still unknown. Further studies are needed to understand the underlying mechanism to improve the utilization of the datasets, as well as to avoid the bias introduced by deliberately choosing datasets.

We would like to point out that there could be other ways to define non-linearity metrics, and the method we proposed here is not necessarily superior to any of these. For example, Emancipator and Kroll (1993) proposed a generic way to measure non-linearity by using the integral of the deviation (L2 norm) of the function from an ideal straight line. However, in this special case (quadratic model), the non-linearity measured using Equation (6) is relatively simple and intuitive. We would also like to mention that it is difficult to calculate statistical significance for the non-linearity term (defined as the ratio of the second order term to the first order term). It is relatively easy to test for differences between the first order coefficients or the second order coefficients alone, but much harder for the ratio, which has a non-Gaussian distribution. Although this is a drawback in our method, defining non-linearity in other ways e.g., using the method Emancipator and Kroll (1993), does not necessarily solve this problem.

## Conclusion

We examined the simultaneous LFP and BOLD recording data and found that the relationship between LFP and BOLD can be non-linear, depending on the type of anesthesia. Under ISO, there is clear evidence not only showing the relationship is non-linear, but also suggesting such non-linearity may come from the non-Gaussian distribution of LFP power. The effect of taking the square to obtain power does not explain all of the non-linearity observed under ISO. Considering the “burst-suppression” phenomenon, which is unique in ISO anesthesia, the switching from “on” and “off” state may induce some non-linearity through a mechanism that is not yet fully understood. This implies that in the future, more generalized methods that do not assume linear dependency might be more desirable than Pearson correlation-based analysis, although, in this particular situation under ISO, the non-linearity has little impact on the Pearson correlation coefficient.

## Data Availability Statement

The datasets analyzed in this manuscript are not publicly available. Requests to access the datasets should be directed to SK, shella.keilholz@bme.gatech.edu.

## Ethics Statement

The animal study was reviewed and approved by The Institutional Animal Care and Use Committee.

## Author Contributions

XZ performed data analysis, developed the theory, and wrote the article. W-JP collected the data. SK supervised the work.

### Conflict of Interest

The authors declare that the research was conducted in the absence of any commercial or financial relationships that could be construed as a potential conflict of interest.
