# Supplementary figures and images for: The Relationship Between Local Field Potentials and the Blood-Oxygenation-Level Dependent MRI Signal Can Be Non-linear
Source: Front Neurosci. 2019 Oct 25;13:1126. doi: 10.3389/fnins.2019.01126 (PMC6823197; doi:10.3389/fnins.2019.01126)

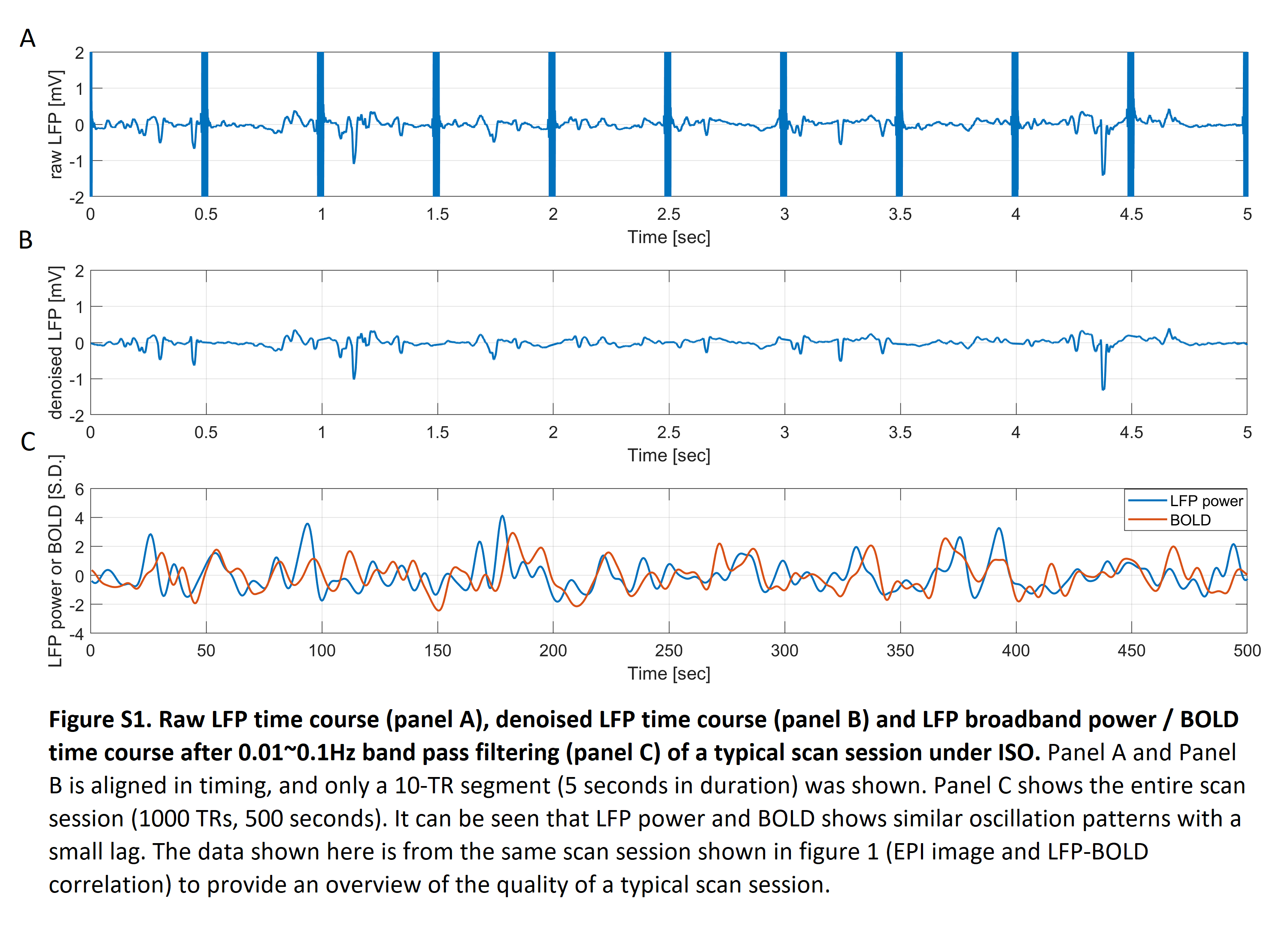

Supplement: Supplementary file 1 [file Image_1.TIF]

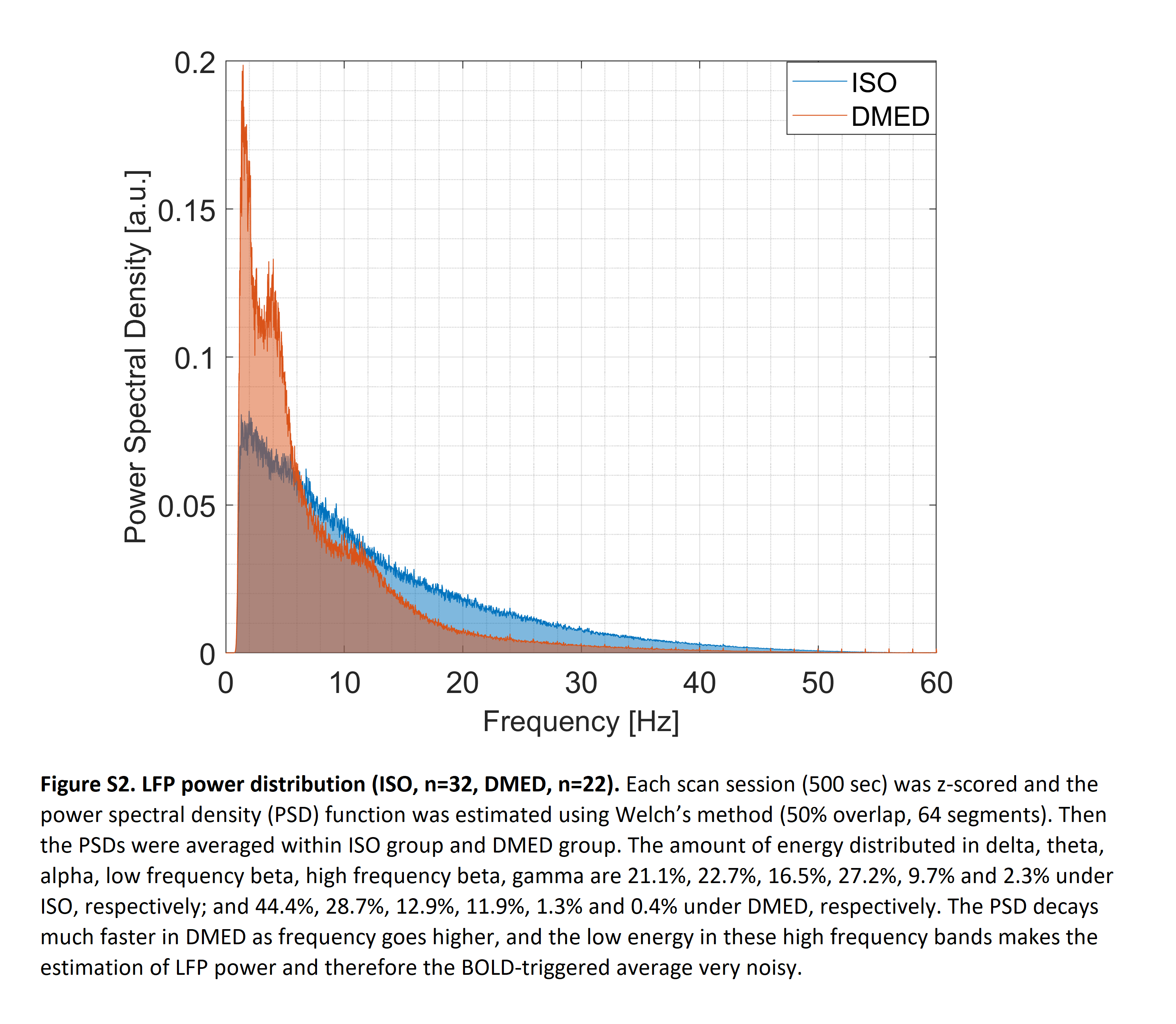

Supplement: Supplementary file 2 [file Image_2.TIF]

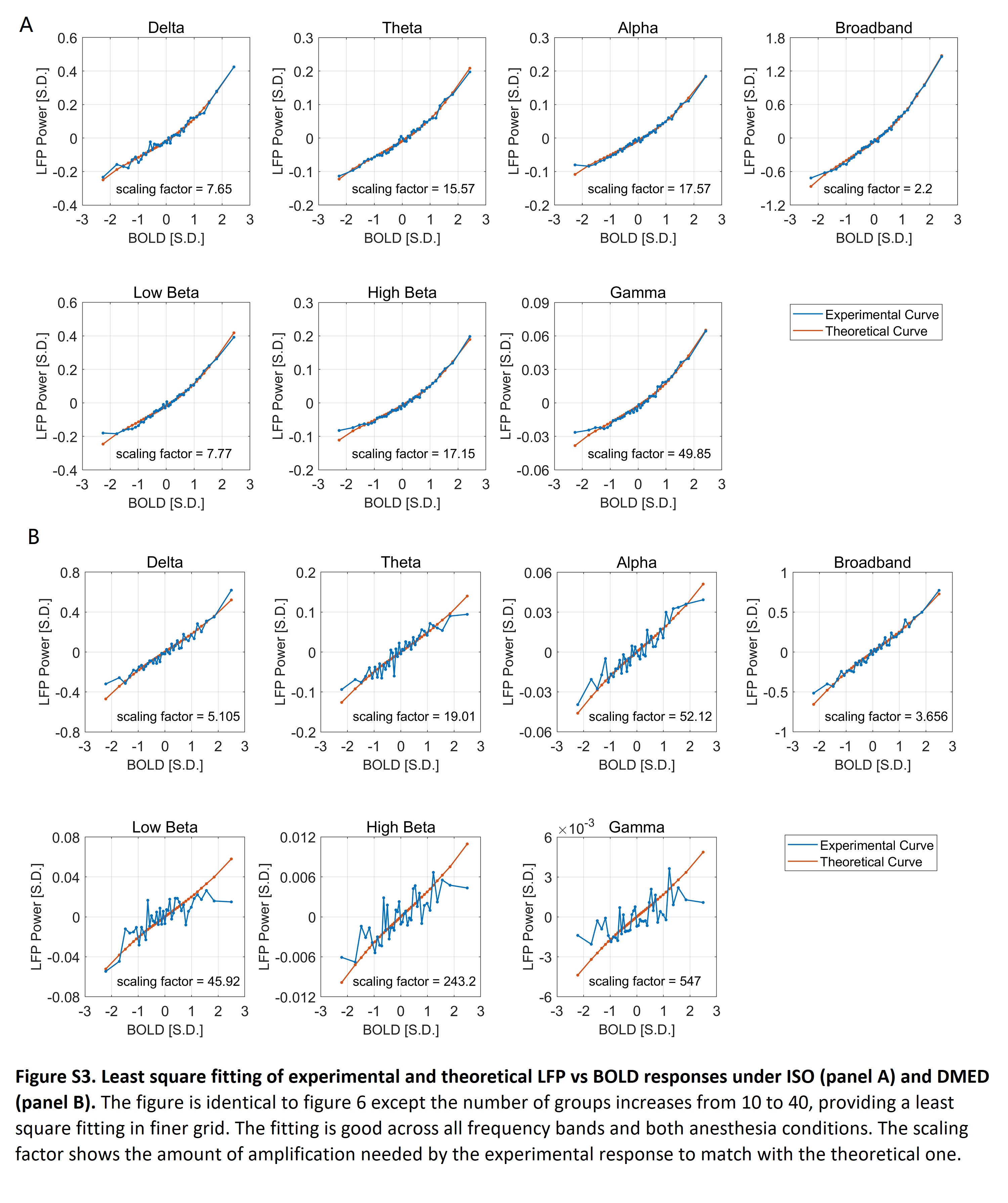

Supplement: Supplementary file 3 [file Image_3.TIF]
